# Overexpression of *ZmIPT2* gene delays leaf senescence and improves grain yield in maize

**DOI:** 10.3389/fpls.2022.963873

**Published:** 2022-07-19

**Authors:** Yongfeng Song, Chunxiang Li, Yong Zhu, Pei Guo, Qi Wang, Lin Zhang, Zhenhua Wang, Hong Di

**Affiliations:** Key Laboratory of Germplasm Enhancement, Physiology and Ecology of Food Crops in Cold Region, Ministry of Education, Northeast Agricultural University, Harbin, China

**Keywords:** maize, leaf senescence, *ZmIPT2* gene, grain yield, overexpression

## Abstract

Cytokinins (CTKs) are a major phytohormone group that are significant in the promotion of cellular division, growth, and divergence. *Isopentenyl transferase* (*IPT*) regulates a rate-limiting step in plant CTK synthesis, promotes the synthesis of isopentenyl adenonucleotides from 5-AMP and isopentenyl pyrophosphate, and then converts both these chemicals into various CTKs. Here, the full-length cDNA of *ZmIPT2*, which encodes 322 amino acids, was isolated and was introduced into a maize inbred line by *Agrobacterium*-mediated transformation. In both controlled environments and field experiments, the overexpression of *ZmIPT2* gene in the transformed plants delayed leaf senescence. Compared to the receptor line, the transgenic maize lines retained higher chlorophyll levels, photosynthetic rates, and cytokinin content for an extended period of time, and produced significantly higher grain yield by a margin of 17.71–20.29% under normal field planting conditions. Subsequently, ten possible genes that interacted with *ZmIPT2* were analyzed by qRT-PCR, showing that the expression pattern of *GRMZM2G022904* was consistent with *ZmIPT2* expression. Through comprehensive analysis, we screened for transgenic lines with stable inheritance of *ZmIPT2* gene, clear functional efficiency, and significant yield improvement, in order to provide theoretical basis and material support for the breeding of new high-yield transgenic maize varieties.

## Introduction

Senescence is a degradative process of plant tissue structures (Rivero et al., 2007). To improve overall plant health, leaves should be engineered for delayed senescence to permit the capture of sunlight energy for a longer duration, allowing for more efficient transformation of light into photosynthates (Sykorova et al., 2008). Additionally, a delay in senescence would also allow tissues to degenerate more slowly. In doing so, the stored resources can be systematically released to the appropriate sink tissues. Advantages of deferred senescence involve a better maintenance of photosynthetic rate, raised plant biomass, improved drought tolerance, and higher seed yield (McCabe et al., 2001; Chang et al., 2003; Rivero et al., 2010).

Cytokinins (CTKs) were thought to have evolved to coordinate the endogenous developmental processes and adaptive responses in plants (Li et al., 2016). Several links have been established between CTK level, pool strength, and source-pool conversion by regulating photoassimilate distribution (Brenner and Cheikh, 1995; Roitsch and Rainer, 2000; Kuiper, 1993). It is generally believed that the CTK-mediated delay of leaf senescence was related to sink/source regulation, and that part of this relationship is mediated by the cell wall invertase (CWINV) protein (Roitsch and Rainer, 2000). Scientists found that leaf senescence was delayed and expression level of CWINV was increased in *PSAG12:IPT* transgenic tobacco, indicating that CWINV was closely related to senescence. The CWINV inhibitor protein was expressed under the CTK inducible promoter, and there was no delay in leave senescence after CTK treatment (Balibrea Lara et al., 2004), indicating that the increased presence of CWINV was a necessary condition for CTK to delay senescence. This, at least, partially explained the molecular mechanisms of CTK in delaying leaf senescence (Peng et al., 2021).

The function of CTKs in deferring leaf senescence has been reported for several plant species (Gan and Amasino, 1995; Robson et al., 2004; Rivero et al., 2007; Sykorova et al., 2008; Peleg et al., 2011; Kant et al., 2015). Gan and Amasino were the first to apply the senescence-specific promoter, SAG12, fused with the IPT gene to explore the function of CTKs in deferring leaf senescence. The transgenic tobacco plants that were created in these studies reported higher biomass and yield (Gan and Amasino, 1995). Further studies on transgenic tobacco found that the expression of the phosphoenolpyruvate gene was regulated by P_SARK_, a gene specifically induced under plant aging and stress conditions (Delatorre et al., 2012). Regulation of the phosphoenolpyruvate gene could maintain a high concentration of cytokinin in plants under water stress, and improve the tolerance of plants to water stress and prolong the photosynthetic life of plant leaves (Rivero et al., 2007). Decreased local CTK production in roots or leaves, which are typically plentiful sources of CTKs, were assumed to initiate leaf senescence (Singh, 1992; Hirose et al., 2008; Kudo et al., 2010). Isopentenyladenine (iP), and one of its glucosides, iP9G, were both capable of delaying chlorophyll degradation in detached cotyledons senescence assays (Hallmark and Rashotte, 2020).

From an agronomic point of view, several studies link increased CTKs levels to seed yield in rice, soybean, and maize. In some varieties of rice, the cytokinin oxidase gene (*OsCTkx2*) was associated with the quantitative trait locus (QTL) for grain production (Ashikari et al., 2005). In soybean, CTKs have been shown to play a significant function on pod growth and flowering (Huff and Dybing, 1980; Ghiasi et al., 1987; Wiebold and Panciera, 1990; Westgate and Peterson, 1993; Nagel, 2001). In maize, CTKs’ exogenous application has been similarly revealed to increase the yield stability of heat stressed plants (Cheikh and Jones, 1994). Therefore, CTKs are involved in a large amount of quantitative and qualitative components of yield. Their participation in a huge quantity of regulatory roles in plants give much incentive to unravel biosynthesis of CTKs and signal pathways (Kieber and Schaller, 2014).

The production of isopentenyl transferase (*IPT*), a key enzyme of CTK biosynthesis, catalyzes the first step of *de novo* synthesis of cytokinin, promotes the synthesis of isopentenyl adenonucleotides from 5-AMP and isopentenyl pyrophosphate, and then converts both these chemicals into various CTKs. The *IPT* gene was first identified in *Agrobacterium tumefaciens* (Gan and Amasino, 1995). Since the discovery of the *IPT* gene, there have been a large number of reports using transgenic technology to study the use of this gene to improve yield, anti-aging, and disease resistance (Noh and Amasino, 1999). The mechanism of inducing the *IPT* gene expression to produce endogenous CTK to delay leaf senescence may be related to promoting the accumulation of stress response related proteins and antioxidant enzymes (Peng et al., 2021). Studies have shown that the heterologous expression of *IPT* under the transcription control of senescence -associated receptor-like kinase (SARK) promoter in wheat can improve plant drought tolerance by delaying cell senescence. Overexpressing *IPT* showed a water tolerance phenotype in different environments: transgenic TR1 and TR4 wheat showed delayed senescence and increased yield under sufficient water conditions (Beznec et al., 2021), similar to the reported effect of *IPT* expression in rice, tobacco, and maize (Rivero et al., 2007; Peleg et al., 2011; Qin et al., 2011; Decima Oneto et al., 2016). Interestingly, in transgenic cauliflower containing the *IPT* gene, a tetrapeptide-like repeat was found, which could induce protein folding, SOD and stress response. The protein folding and carbon fixation related protein levels of this transgenic cauliflower were higher than those of non-transgenic cauliflower, and the iron SOD and APX activities were also significantly higher than those of non-transgenic cauliflower (Liu et al., 2011). The transgenic plants had an increased number of stress-related proteins and molecular chaperones, induced the expression of stress-related genes, and protected cells during aging in contemporary plant breeding programs (Peng et al., 2021).

These techniques are considered new strategies for engineering CTKs for better crop production in agricultural systems (Jameson and Song, 2016; Kieber and Schaller, 2018). In addition, *Arabidopsis* researchers have shown that the manipulation of CTK levels via *IPTs* can enhance salt stress tolerance and drought (Li et al., 2016), laying the groundwork for similar studies in agriculturally significant plants. In addition, a growing number of studies have shown that during stress, the expression of the *IPT* gene can induce plants to produce various resistant or active substances, which can produce CTK responses, coordinate, and interact with other hormones. The production of various resistance and active substances in plants can enhance photosynthesis. Various metabolites can be synthesized to promote the response of plant cells to various adverse environments or diseases and insect pests. Moreover, agronomic traits have been significantly improved by the induction of the *ITP* gene (Li and Zhao, 2011), which provided a novel base for future molecular breeding of crop plants (Jameson and Song, 2016).

In crops such as lettuce (McCabe et al., 2001), rice (Jin et al., 2002), wheat (Sykorova et al., 2008), and ramie (An et al., 2017), CTK manipulation not only delays the aging of leaves, but also improves agronomic traits and stress resistance of crops. Gan and Amasino transferred the chimeric genes *SAG12* and *IPT* into tobacco and found that the transgenic plants showed higher biomass and yield (Gan and Amasino, 1995). In rice, *IPT*-induced CTKs synthesis reduced environmental stress consequences on photosynthesis and yield and maintained nitrogen (N) acquisition (Reguera et al., 2013). Overexpression of *OsIPT9* in rice can increase the CTK level of caryopsis development, resulting in enhanced grain filling of large and multi-panicle rice varieties, thus increasing yield (Panda et al., 2018). Transgenic wheat containing the *IPT* gene driven by AtMYB2xs promoter had improved yield under both sufficient water and water stress conditions (Joshi et al., 2019). Regulating *IPT* by using the *AtMYB32* promoter can improve rape yield under both drought and conventional irrigation conditions (Kant et al., 2015). Multiple species showed improved yield by increasing *IPT* expression levels under drought conditions, indicating that if the expression of *ITP* can be fully controlled, it could be a key driver of yield in crop systems.

In maize, eight *IPT* genes have been found in the genome, all of which have complete open reading frames (ORFs) except *ZmIPT3*. Among the completed ORFs, the *ZmIPT2* gene encodes a 322 amino acid protein and is stably expressed at the transcriptional level, in addition to being the highest expressed protein (Takei et al., 2001). Some studies have proved that in maize tissue, the content of CTKs was directly proportional to the expression level of *ZmIPT2*. When the expression level of *ZmIPT2* increased, the CTKs content also increased. Therefore, the expression of this gene was considered to be related to the content of cytokinin. The *ZmIPT2* gene played a very important role in overall seed development, from endosperm cell division to embryo development, and played a role in germplasm strength. It can also delay leaf senescence and improve yield (Brugiere et al., 2008).

The endeavor to comprehend the molecular basis of this increase in yield owning to deferred senescence and the possible involvement of CTKs in this process will be useful in aiding breeders and scientists alike. Our laboratory cloned *ZmIPT2* gene from early maize, and transformed the maize using *Agrobacterium*, showing that the transgenic offspring held their leaves during the regular leaf senescence stage, and maintained a brighter green color in their leaves. The transgenic offspring also had an increased yield, although the genetic instability phenomenon was observed. Therefore, we reconstructed the monocotyledonous plant expression vector of this gene, and transformed maize inbred lines by *Agrobacterium tumefaciens* infection of young embryos to obtain T2 transgenic lines. We clarified the basic characteristics of this gene through bioinformatic analysis, examined the expression pattern under different growth stages, and analyzed the physiological indexes and yield traits of transgenic lines. The interacting genes of *ZmIPT2* were then screened by qRT-PCR. The transgenic maize line that we have generated may be of great value as a genetic resource for further high-yield maize breeding.

## Materials and methods

### Plant materials

The maize inbred line Zheng 58 was used to clone the target gene and the maize inbred line C01 was used as the receptor material for genetic transformation. The material for Zheng 58 was obtained from the Maize Research Institute of Northeast Agricultural University. The inbred line C01 used as a transgenic receptor was provided by the Life Science and Technology Center of China Seed Group Co., Ltd. According to the relevant laws of China, after the experiment the seeds were preserved on-site and the plants were properly disposed of.

*Agrobacterium tumefaciens* strain LBA4404 came from our laboratory. *Escherichia coli* strain DH5a came from Beijing Quanshijin company. The basic plant expression vector used in the transformation experiments was pEAST-T1, with the ubiquitin promotor, T-NOS terminator, and the CaMV35S promoter driven bar gene selection marker, which was provided by the Institute of Crop Research, Chinese Academy of Agricultural Sciences. Taq DNA polymerase was purchased from Beijing Quanshijin biological Co., Ltd.

### Methods

#### The characterization and cloning of the *ZmIPT2* gene

##### Bioinformatics analysis of *ZmIPT2* gene

The *ZmIPT2* gene (Gene ID: *GRMZM2G084462*) was localized in bin 2.04 region based on B73 RefGen_v2 genome-wide data. Analysis of the ZmIPT2 ORF was performed with ORF finder^1^. The second-order structure of the ZmIPT2 protein was predicted using SOMPA^2^, with SWISS – MODELL^3^ to predict the protein tertiary structure. The Conserved Domain Database website^4^ was used for protein structure analysis, and Phytozome V 12.1 was used for the other species of IPT family protein sequences. The system evolution tree was constructed by using ClustalX 1.83 and MEGA 5 software, with the bootstrap value adjusted to 1000. The physicochemical properties and hydrophobicity of the ZmIPT2 protein were analyzed with protparam^5^ and ProtScale^6^. The subcellular localization of ZmIPT2 protein was predicted using Softberry^7^. The *cis*-acting elements of the *ZmIPT2* gene flanking sequences were identified using the PlantCARE program^8^.

##### Gene cloning and plant expression vector construction

Using the accession number EU263126.1 on GenBank, the mRNA sequence of *ZmIPT2* gene was downloaded and PCR primers were designed. Total RNA was extracted from leaves of maize inbred line Zheng 58 with the plant RNA Kit (Beijing Tiangen, China) and reverse transcribed to obtain the first strand cDNA. Under the following conditions, the cDNA was amplified by PCR with specific primers of *ZmIPT2* using KOD plus NEO (Toyobo) polymerase mixture. The recovered PCR products were sent to Shanghai Sangong for sequencing.

Total RNA was extracted from leaves of the inbred maize line Zheng 58 with the Plant RNA Kit (Beijing Tiangen, China) following the manufacturer’s protocols. Extracted RNA was treated with DNase I (Omega, GA, United States) to remove residual DNA. The first-strand cDNA was synthesized from 1 μg of total RNA using 1 μL (200 U) PrimeScriptTM RT Enzyme Mix I (TaKaRa) according to the manufacturer’s protocol, to produce the full-length cDNA of *ZmIPT2*. PCR was performed on the cDNA using specific primers for *ZmIPT2* using the KOD-Plus-Neo (TOYOBO) polymerase mix under the following conditions. The amplification product was purified from 1.0% agarose gel and cloned into the pEASY-T1 Simple vector (Transgen) to be sent for sequencing. The primers are listed in Supplementary Table 1. The full sequence of the *ZmIPT2* cDNA clone used in this study can be found in GenBank (GenBank Accession No. EU263126.1).

For generating the transgenic lines overexpressing *ZmIPT2*, the coding sequence of *ZmIPT2* was subcloned from the pEAST-T1 vector into the pEC-Ubi-Tnos-Bar vector, which consisted of a maize constitutive ubiquitin promoter, Bar gene with the CaMV 35S promoter, and a Tnos terminator (Sambrook et al., 1982), using to the standard protocol for the In-Fusion Cloning Kit [Takara Biomedical Technology (Beijing) Co., Ltd]. The recombinant plasmid was finally transformed into *Agrobacterium tumefaciens* LBA4404 using the freeze-thaw method (Supplementary Figure 1).

##### Maize transformation

In this study, maize inbred line C01 was used as the starting genetic material. Maize transformation was performed according to the method published by Li et al. (2008). C01 seeds were surface sterilized and cultured on sterile MS agar at 28°C until etiolated seedlings grew to 3.0–4.0 cm. The maize shoot tip with exposed meristem was immersed in the transformed *Agrobacterium* suspension, which was grown a logarithmic stage, and then diluted to an OD600 of 0.8. The buds were soaked at 0.05 MPa for 10 min and then co-cultured on modified MS agar in the dark at 24°C for 3 days. The seedlings were then transplanted into pots and transferred to the greenhouse to continue to grow. Transgenic seedlings sprayed with 0.1% herbicide Basta were detected by PCR, and seeds were harvested from positive plants (T0). T1 plants were grown from harvested seeds and self-pollinated to produce the T2 generation. Because of the vector contains a screening marker gene Bar gene, the positive transgenic plants of each generation had the integration and stability of the *ZmIPT2* gene by verified by PCR and Bar protein test strip. The expression level of *ZmIPT2* and copy number in transgenic plants was confirmed by qRT–PCR.

#### Molecular detection of transgenic maize lines

##### PCR and RT-PCR

The leaf of DNA was extracted by CTAB method (Murray and Thompson, 1980), and specific primers were designed according to the target gene sequence and terminator sequence. One end of the primer was located inside the target gene, and the other end was located inside the terminator. The primers of *ZmIPT2* gene were F (5′-TTTAGCCCTGCCTTCA-3′), R (5′-AACCCATCTCATAAATAACG-3′), and bar gene specific primers BF1 (5′-CCATCTCAACCACTACATCG-3′) and BR1 (5′-AGCTGCCAGAAACCCCACGT-3′). PCR reactions were set up in volumes of 20 μL, as recommended by the manufacturer. The thermal cycling conditions were 95°C for 5 min, followed by 30 cycles of 95°C for 30 s, 50°C for 30 s, 72°C for 70 s, and 72°C for 10 min. Total RNA was extracted using Trizol reagent (Tiangen, Beijing, China) from 100 mg of maize seedling leaves. The RNA was DNase-treated, and 500 ng of this treated RNA was used for inverse transcription with the RT Reagent Kit (Quanshijin, Beijing, China), following the manufacturer’s protocols. The generated cDNA samples were diluted fivefold to serve as templates for the subsequent PCR. The primers Actin-F (5′-GTTGGGCGTCCTCGTCA-3′) and Actin-R (5′-TGGGTCATCTTCTCCCTGTT-3′) were designed for qRT-PCR, and three technical replicates were used for each of the RT-PCR experiments in this research.

##### Expression analysis by real-time quantitative PCR

The V6 (sixth leaf), R1 (silking), R2 (blister stage), and R6 (physical maturity) leaves of maize were collected at different growth stages. The Trizol reagent (Quanshijin, Beijing, China) was used to extract total RNA. For each sample, the Transscript^®^ One step gDNA Removal Kit mix (Transgen Biotech, Beijing, China) was combined with 500 ng RNA to synthesize cDNA. qRT-PCR was performed using the TransStart Tip Green qPCR SuperMix fluorescent quantitative Kit (Genetically Modified Biotechnology Company, Beijing, China). The Actin1 and *ZmIPT2* gene-specific primer sequences used for qRT-PCR are located Supplementary Table 1. The gene expression levels were calculated using the 2–ΔΔCt formula method (Livak and Schmittgen, 2001). For each sample, three biological replicates were included, with three technical replicates for each. The subsequent analysis was performed by using the obtained qRT-PCR data.

##### The copy number analysis of transgenic plants

The transgene copy number was detected by qRT-PCR. Actin1 (Gene ID: 100282267), which is a single copy gene in maize, was used as the endogenous reference gene. According to the principle that the CT value obtained by PCR reaction is linearly inversely proportional to the logarithm of the number of initial templates, a quantitative standard curve with a correlation coefficient of more than 0.99 between the internal reference gene actin and the target gene *ZmIPT2* was prepared. After diluting the DNA concentration of each sample to be tested by five times, the qRT- PCR reaction was conducted to obtain CT values and calculate the number of initial templates of each sample according to the standard curve. Since the actin gene is a single copy in maize, the ratio of the logarithm of the initial template number of *ZmIPT2* gene and actin gene is the copy number of the target gene in maize (Han et al., 2016).

#### Detection of physiological and biochemical identification of transgenic maize lines with *ZmIPT2* gene

The relative chlorophyll content, photosynthetic efficiency, and dynamic changes of cytokinin content in different growth stages of the transgenic maize lines and receptor control lines with high target gene expression were quantified.

To measure photosynthetic efficiency, photosynthetic rate was measured using a Li-6400 portable photosynthetic system (Li COR, Lincoln, NE, United States). In order to reduce the laboratory environment and the gradient in the tube of the gas exchange system, the concentration of CO2 in the sample was set to 500 μmol^–1^, the relative humidity in the tube was set to 65%, and the temperature of the blade in the tube was set to 28°C. Conventional methods were used to estimate outdoor *in situ* gas exchange between 8:30AM and 11:30AM. The environmental conditions in the colorimeter were as described above and were set prior to recording the gas exchange data. No less than 20 min was required to achieve a stable gas exchange measurement, i.e., photosynthetic rate change < 0.5% in 1 min.

To measure chlorophyll content, leaves [100 mg fresh weight (FW)] were incubated in 10 ml tubes containing 5 ml 80% acetone at room temperature in darkness until the tissues turned white. Total chlorophyll content was set by measuring the absorbance at 645 and 663 nm applying the formula 20.2 A_645_ + 8.02 A_663_ (Chory et al., 1994). The extraction and purification of isopentenyl adenine standard (IP) and zeatin in leaves to measure the dynamic changes of cytokinin content were performed according to of the protocol described by Dong et al. (2008). The cytokinin content was decided by enzyme-linked immunosorbent assay (ELISA) protocol (Wu et al., 1988). Three samples were taken from each line in each period for mixed treatment, and three technical replicates were performed for each sample.

#### Field experiment of transgenic maize lines

The seeds of transgenic plants and recipient inbred line C01 were planted in the transgenic experimental practice base of Northeast Agricultural University. The field experiment was performed by using the comparison method. The row spacing for each plot was 65 cm, the length was 2.0 m, and the plant spacing was 2 cm. The agronomic characteristics of each plot during maize growth were considered similar to a conventionally managed field. Three consecutive plants were selected in the middle row of each plot to avoid the effects of the border plants, and the identification method of agronomic traits was adopted from the Description and Data Standard of Maize Germplasm Resources. The agronomic traits in field investigation included ear length, ear diameter, axis diameter, grain length, grain width, grain thickness, bare tip length, 100-grain weight, and plot yield. Excel statistical data was used to test agronomic traits and green-holding traits, SPSS 22.0 was used for the analysis of data processing, and the *T*-test was used to test the significance of data difference.

#### *ZmIPT2* interaction gene analysis

The amino acid sequence of the *ZmIPT2* gene was submitted to STRING^9^ to predict the proteins that interacted with the putative ZmIPT2 protein. These gene sequences were downloaded from NCBI database. The primers for these proteins coding sequences were designed using Primer5 and are listed in Table 3. The relative expression of these interacting proteins in maize leaves at different growth stages were verified by RT-qPCR. Then, the interacting genes were further determined by bioinformatic analysis.

**TABLE 1:** Amino acid compositions of *ZmIPT2.*

Types	Percentage (%)	Name	Quantity (%)
Hydrophobic amino acids	51.60	Ala	16.10
		Ile	3.10
		Leu	7.50
		Met	1.60
		Phe	3.10
		Pro	3.70
		Asn	2.20
		Val	12.70
		Trp	1.60
Hydrophilic amino acids	37.90	Asp	0.053
		Cys	0.006
		Glu	9.90
		Gly	8.70
		Ser	2.50
		Thr	5.30
		Tyr	1.20
		Gln	1.60
Basic amino acids	13.30	Arg	7.10
		His	2.80
		Lys	3.40
Acidic amino acids	15.20	Asp	5.30
		Glu	9.90

**TABLE 2:** Copy number of *ZmIPT2* gene in trangenic lines.

Line	*Actin*		*ZmIPT2*			Copy number
	Ct value	Amount of template	Ct value	Amount of template	Ratio to *Actin*	
						
C01	25.88	0.270	28.98	0	0	0
DNIPT2-C14	23.83	0.042	29.54	0.036	0.86	1
DNIPT2-C33	20.10	0.559	24.51	0.458	0.82	1
DNIPT2-C34	20.12	0.570	25.07	0.719	1.26	1

**TABLE 3:** The survey results of yield related traits of the transgenic maize lines with *ZmIPT2* gene.

Transgenic	Line	Ear height (cm)	Ear diameter (mm)	Cob diameter (mm)	Bald tip length (cm)	Grain length (mm)	Grain width (mm)	Grain thickness (mm)	100-grain weight (g)	Yield per plot (kg)	Yield plot increased
T2	C01	13.87 ± 0.29	46.45 ± 0.04	29.08 ± 1.62	0.00 ± 0.00	10.51 ± 1.11	9.32 ± 0.48	4.95 ± 0.58	30.41 ± 3.09	5.34 ± 0.03	
	DNIPT2-C14	15.20 ± 0.75**	45.33 ± 0.11	29.95 ± 1.20	0.57 ± 0.06*	11.49 ± 1.25*	9.11 ± 0.51	5.04 ± 0.20	30.80 ± 0.01	5.59 ± 0.01**	4.68%
	DNIPT2-C33	13.90 ± 0.70	49.17 ± 0.65*	31.70 ± 0.82	0.00 ± 0.00	11.48 ± 1.57*	8.09 ± 0.88	5.76 ± 0.62**	33.58 ± 0.00**	5.82 ± 0.00**	8.99%
	DNIPT2-C34	15.83 ± 0.64**	46.78 ± 1.74	32.23 ± 3.79*	0.47 ± 0.31	11.41 ± 0.35*	7.82 ± 0.24	5.24 ± 0.52	31.68 ± 0.00	5.66 ± 0.00**	5.99%
T3	C01	12.93 ± 0.33	42.61 ± 1.84	30.16 ± 0.66	0.00 ± 0.00	10.31 ± 0.19	8.51 ± 0.22	4.70 ± 0.46	27.32 ± 0.47	4.09 ± 0.24	
	DNIPT2-C14	17.23 ± 0.78**	44.80 ± 1.07**	27.97 ± 1.11*	0.83 ± 0.46**	10.36 ± 0.08	9.22 ± 0.35	5.29 ± 0.16	32.10 ± 0.08**	4.92 ± 0.20**	20.29%
	DNIPT2-C33	13.47 ± 0.52	45.71 ± 0.31**	31.19 ± 0.63	0.73 ± 0.23*	9.69 ± 0.33	7.92 ± 0.32	5.45 ± 0.48	31.20 ± 0.37**	4.79 ± 0.08**	17.11%
	DNIPT2-C34	14.90 ± 0.35*	48.58 ± 0.59**	33.25 ± 1.27	0.17 ± 0.17	10.20 ± 0.24	8.35 ± 0.15	5.81 ± 0.28	31.10 ± 0.78**	4.90 ± 0.08**	19.80%

Plants were grown in the molecular breeding experimental base at Northeast Agricultural University (Harbin, China) in a randomized comparative trial with three replications. The row spacing was 65 cm, the length was 2.0 m and the plant spacing was 2 cm. The results are presented as mean ± SD (n = 3). Asterisk (**) indicates extremely significant differences at P\0.01 according to Student’s t-test. Asterisk (*) indicates significant differences at P\0.05 according to Student’s t-test.

## Results

### The characterization and cloning of the *ZmIPT2* gene

**FIGURE 1:**
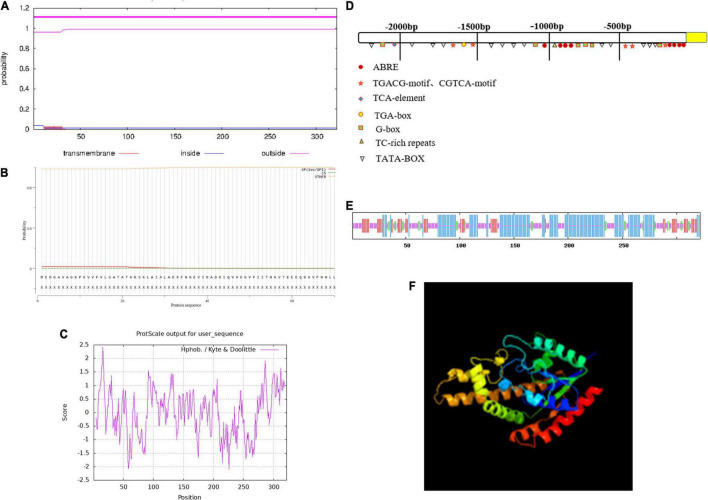
The characterization of the ZmIPT2 protein. **(A)** Transmembrane domain prediction of ZmIPT2. **(B)** Prediction of signal peptide and splice siteS. **(C)** Hydrophobicity analysis of ZmIPT2 coding protein. **(D)** Analysis of flanking sequence of *ZmIPT2* gene. **(E)** Secondary structure analysis of the ZmIPT2. The helix (h), extended strand (e), coil(c), and turn (t) are indicated in different color as blue, red, yellow, and green, respectively. **(F)** 3-D structure model of ZmIPT2.

The *ZmIPT2* gene encoded a putative protein of 322 amino acids with a predicted molecular mass of 34.49 kDa, and a predicted isoelectric point (PI) of 5.11. The amino acid sequence comparison of the ZmIPT2 protein via ExPASy Protparam is shown in Table 1. Subcellular localization predictions showed that the protein was most likely to be localized on the cytoplasm with a confidence level of 0.478 and may also be distributed in the nucleus and mitochondria but with lower confidence (Supplementary Table 1). The results of cross-membrane domain analysis found that the ZmIPT2 protein did not exist in the transmembrane domain and was located in the extramembrane region with the greatest probability (probability = 1.1), indicating that the ZmIPT2 protein did not carry out transmembrane transport, but directly exercised its function, and that it belonged to the non-transmembrane protein class (Figure 1A). The results of signal peptide analysis showed that there was no signal peptide in the protein sequence. The ZmIPT2 protein was not a secretory protein and therefore could not migrate in cells (Figure 1B). Hydrophobic structure analysis showed that the ZmIPT2 protein contained a typical hydrophobic region at about 16 amino acids (Figure 1C). Meanwhile, the *ZmIPT2* promoter indicated that it had core elements such as a TATA box and a CAAT box, as well as a large number of abiotic stress related elements referred to such as ABRE, an auxin response element, and a *cis*-acting regulatory CAT box that was involved in meristem expression (Figure 1D). It is speculated that the *ZmIPT2* promoter may be a stress-inducible promoter. Its predicted Secondary Structure includes alpha-helices (55.85%), random coils (31.06%), extended strands (9.90%), And β-helices (7.76%) (Figure 1E). Its three-dimensional homology model is a 3A8T. 1 protein sequence with a similarity of protein 40.58% (Figure 1F).

The CD search results indicated that the ZmIPT2 protein contained a conserved P-loop NTPase domain (Supplementary Figure 2). A BLAST search of the NCBI protein database showed that the predicted amino acid sequence of ZmIPT2 had different degrees of similarity with other known proteins. IPT protein sequences of 35 different species were downloaded, including those of *Sorghum bicolor, Panicum miliaceum, Panicum hallii*, and *Dichanthelium oligosanthes* (Supplementary Figure 3A). The results of the constructed evolutionary tree showed that ZmIPT2, SbIPT3, and DoIPT3 were in the same branch, among which ZmIPT2 and SbIPT3 had the closest genetic relationship with an amino acid consistency of 86.18% (Supplementary Figure 3B). The evolutionary grouping of proteins may reveal similarities and differences in function.

RT-PCR was performed using RNA from the maize line C01 as template for the synthesis of cDNA. The amplified cDNA fragment showed a 969-bp band in a 1% agarose gel and was ligated into the pEasy-T1 Simple vector. Sequencing showed that the plasmid contained the same sequence as the candidate gene in MaizeGDB^10^. The reconstructed full length of the cDNA sequence, which was 1339-bp long, was identified as the sequence of the full-length *ZmIPT2* gene, including the 969 bp CDS, 155 bp 5′-UTR, and 216 bp 3′-UTR (Supplementary Figure 4).

### Molecular detection of transgenic maize lines

Over 30 T0 independent transgenic plant lines overexpressing the *ZmIPT2* gene from *Agrobacterium tumefaciens* were established and propagated in the greenhouse. The primer pair *ZmIPT2-F*/*ZmIPT2-R* was shown to specifically recognize transgenic maize *ZmIPT2* by PCR amplification (Supplementary Figures 5A,B). Three transgenic maize lines (DNIPT2-C14, DNIPT2-C33, and DNIPT2-C34) with a single ZmIPT2 integration event were verified by copy number analysis of the T3 generation (Table 2).

**FIGURE 2:**
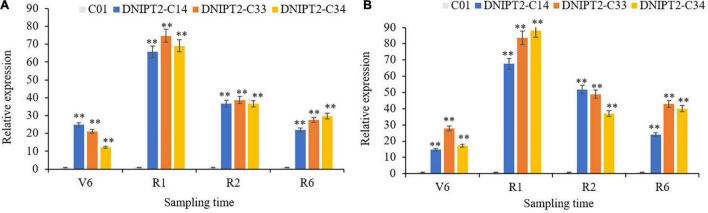
Relative expression levels of transgenic *ZmIPT2* gene in maize leaves at different stages in T2 and T3 generation. **(A)** Shows Relative expression levels of transgenic *ZmIPT2* gene in maize leaves at different stages in T2 generation, V6 (sixth leaf), R1 (silking), R2 (blister stage) and R6 (physical maturity); **(B)** shows Relative expression levels of transgenic *ZmIPT2* gene in maize leaves at different stages in T3 generation. The error bars denote standard deviations of the qRT-PCR signals (*n* = 3). There was significant difference or extremely significant difference in the expression level of ZmIPT2 (**P < 0.01, Student *t*-test).

Quantitative real-time PCR (qRT-PCR) was used to monitor the expression of *ZmIPT2* in the leaves of transgenic lines at different growth stages (Figures 2A,B). The overexpressed *ZmIPT2* gene first increased and then decreased with the development of the maize plants. The relative expression level of the gene reached its peak at the R1 stage, and then decreased in the following stages. The stability of *ZmIPT2* in T2 and T3 plants was monitored by RT-PCR. No expression of the *ZmIPT2* gene was detected in receptor line C01 (Supplementary Figures 5C,D).

### The overexpression of the *ZmIPT2* gene delayed the senescence of maize leaves

**FIGURE 3:**
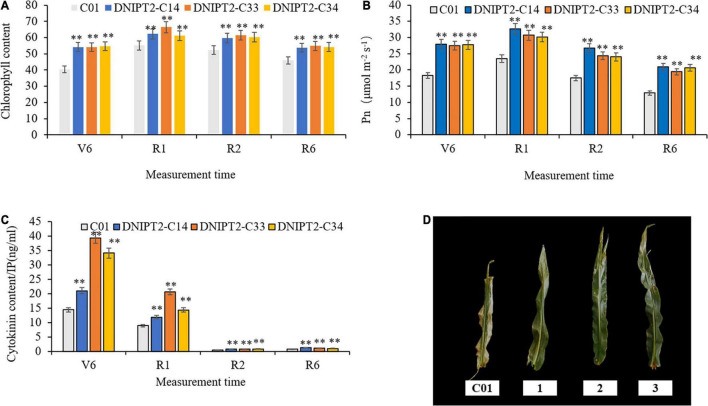
Detection of physiological and biochemical indexes of transgenic maize lines with *ZmIPT2* gene. **(A)** Shows the relative chlorophyll content of transgenic lines at different growth stages of maize, V6 (sixth leaf), R1 (silking), R2 (blister stage) and R6 (physical maturity); **(B)** shows the photosynthetic rate content of transgenic lines at different growth stages of maize, V6 (sixth leaf), R1 (silking), R2 (blister stage) and R6 (physical maturity); **(C)** shows the content of cytotaxon (CTK) of transgenic lines at different growth stages of maize, V6 (sixth leaf), R1 (silking), R2 (blister stage) and R6 (physical maturity). **(D)** The comparison of leaf greenness in T3 transgenic maize lines with *ZmIPT2* gene at generation at mature stage, C01, negative control; 1, DNIPT2-C14; 2, DNIPT2-C33; 3, DNIPT2-C34. There was significant difference or extremely significant difference in the expression level of *ZmIPT2* (**P < 0.01, Student *t*-test).

The most direct manifestations of maize leaf senescence are a decrease in photosynthetic rate and a decrease in chlorophyll content. Chlorophyll content was recorded at multiple growth stages, and the data showed that the chlorophyll levels in the transgenic lines were significantly higher in the four growth stage of the transgenic line compared to the receptor line. The transgenic plants maintained better canopy coverage and higher chlorophyll levels than recipient plants. In addition, the relative chlorophyll content of transgenic lines was the lowest at V6 (jointing stage), reached the peak at R1 (flowering stage), then decreased slowly for the remainder of the plants’ life (Figure 3A).

It was found that among the maize plants, the transgenic lines showed a large green leaf area and obvious “stagnant green” phenomenon, while the receptor line showed a greater proportion of leaf edges withered and turned yellow (Figure 3D), thus proving that the overexpression of *ZmIPT2* gene postponed the process of leaf senescence. In addition, when compared with the receptor line, the photosynthetic rate of the three transgenic lines had significantly increased by 22.16–39.56% in the four growth stages. With the process of leaf senescence, the photosynthetic efficiency of all lines showed a downward trend after flowering. Compared with the receptor control, the photosynthetic efficiency of the transgenic lines decreased more slowly, maintained high photosynthetic efficiency for a longer time, and the plants accumulated more photosynthetic products (Figure 3B).

The CTKs content in all plants reached its highest at growth phase R1, and then began to decrease (Figure 3C). In R6, the CTKs content of the receptor line was 0.81. The CTKs content of DNIPT2-C14, DNIPT2-C33, and DNIPT2-C34 were significantly higher than that of C01 (*P* < 0.01) at 1.29, 1.18, and 1.07, respectively. Although the CTKs content of the transgenic line was lower than that of the first three stages, they were still significantly higher than that of the receptor line C01, indicating that the overexpression of *ZmIPT2* gene in the transgenic strain resulted in the increase of endogenous CTKs content in leaves and delayed the decline rate of CTKs.

### Over-expressing of *ZmIPT2* gene increased grain yield

**FIGURE 4:**
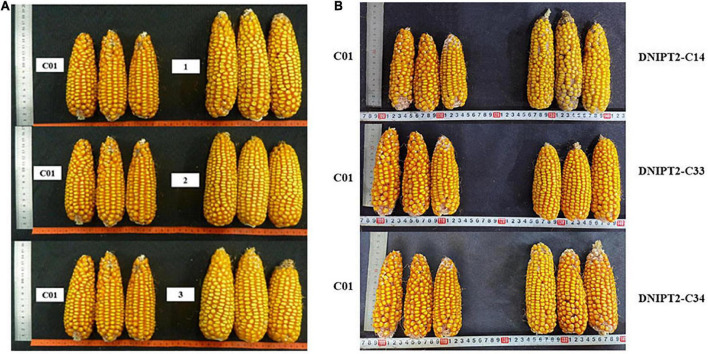
The ear and grain of transgenic maize lines with *ZmIPT2* gene and the receptor line C01. **(A)** The ear of T2 transgenic maize lines with *ZmIPT2* gene and the receptor line C01. **(B)** The ear of T3 transgenic maize lines with *ZmIPT2* gene and the receptor line C01. C01, negative control; 1, DNIPT2-C24; 2, DNIPT2-C33; 3, DNIPT2-C34.

The important agronomic traits and yield-related traits of T2 and T3 transgenic lines were quantified (Table 3). Compared with the recipient control line, the ear length, ear thickness, grain length, grain width, and grain thickness of the transgenic lines increased significantly (Figure 4). The average plot yield of T2 and T3 transgenic plants under normal field planting conditions ranged from 12.49 to 13.05%, with the yield of the DNIPT2-C14 line as the highest. Although the important agronomic traits of the three transgenic strains were improved compared with the recipient control line C01, the difference was not statistically significant, indicating that the introduction of *ZmIPT2* gene had no adverse effect on the growth and development of maize plants.

### *ZmIPT2* interaction gene analysis

**FIGURE 5:**
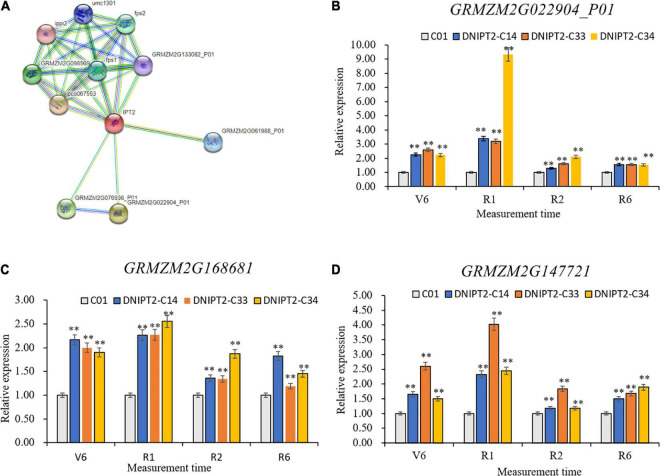
Gene analysis of ZmIPT2 interaction. **(A)** The predictive and analysis of *ZmIPT2* interaction protein. **(B)** Relative expression levels of transgenic *GRMZM2G022904_P01* gene in maize leaves at different stages. **(C)** Relative expression levels of transgenic *GRMZM2G168681* gene in maize leaves at different stages. **(D)** Relative expression levels of transgenic *GRMZM2G147721* gene in maize leaves at different stages. There was significant difference or extremely significant difference in the expression level (**P < 0.01, Student *t*-test).

Ten proteins were predicted to interact with *ZmIPT2* (Figure 5), including *GRMZM2G022904*, *GRMZM2G076936*, *GRMZM2G168681*, *GRMZM2G06198*, *GRMZM2G133082*, *GRMZM2G14772*, *GRMZM2G10828*, *GRMZM2G145029*, *GRMZM2G098569*, and *GRMZM2G027059*. *GRMZM2G022904* and *GRMZM2G076936* were cytochrome P450 superfamily proteins, which participate in the decomposition and anabolism of plant hormones. The other interacting proteins were involved in related reactions of *IPT*. qRT-PCR showed that the temporal and spatial expression patterns of *GRMZM2G168681*, *GRMZM2G147721*, and *GRMZM2G022904* in transgenic lines were consistent with those of *ZmIPT2* gene and reached their maximum at the flowering stage (Figures 5A–C). *GRMZM2G022904*, *GRMZM2G168681*, and *GRMZM2G147721* were localized in bin 7.02, 8.03, and 8.06, respectively, in regions based on B73 RefGen_v2 genome-wide data analysis. The CD-Search results indicated that their protein contains conserved P450 superfamily and isoprenoid biosynthesis superfamily sequences (Supplementary Figure 6). In addition, *GRMZM2G022904* encoded a cytokinin hydroxylase, which can participate in the regulation of isoprenoid CK and affect the development process of plants. However, *GRMZM2G168681* and *GRMZM2G147721* have not been fully proven to interact with the *ZmIPT2* gene. The results of qRT-PCR, bioinformatics, and research reports preliminarily determined that the *GRMZM2G022904* gene may interact with *ZmIPT2* gene to participate in the molecular regulation mechanisms of anti-aging and increasing yield of maize.

## Discussion

### Importance of cytokinins in plant senescence

Cytokinins are known to play an important function in controlling leaf senescence and increasing grain yield of plants. There are two main approaches to regulating endogenous CTKs, which are utilized by up regulating or down regulating two kinds of genes. One of these genes encodes for *IPT*, which is the rate-limiting enzyme in CTK synthesis. The other is CTK oxidase/dehydrogenase, which are related to its degradation. These genes can oxidize the side chain groups of CTKs and irreversibly inactivate them.

In rice, the number of grains was increased by the accumulation of CTKs in the inflorescence meristem per panicle. Rice gene *Gn1a* encodes an oxidase/dehydrogenase (*OsCKX2*), which plays a role in the degradation of CTKs. The 11 bp deletion in the coding region of *OsCKX2* resulted in its early termination, which reduced the expression of *OsCKX2* and increased the number of grains (Ashikari et al., 2005). In wheat, the direct homolog of rice *OsCKX2*, *TaCKX6-D1*, was correlated with 1000-grain weight significantly through association analysis and linkage mapping (Zhang et al., 2012). Therefore, the regulation pathway of CTKs may play a crucial role in grain yield.

In general, leaf senescence is accompanied by the degradation of chlorophyll, proteins, and nucleic acids, as well as a decrease in photosynthetic rate. CTKs can lessen sugar accumulation, increase chlorophyll synthesis, and prolong the leaf photosynthetic period (Wu et al., 2021). Many studies have shown that the *IPT* gene plays an important role in the regulation of endogenous CTK activity required for cell division (Qin et al., 2011) and can delay leaf senescence. Guo and Gan (2011) found that the expression of the *IPT* gene at axillary bud sites was increased in *Arabidopsis* mutant MYB2, resulting in the increase of the CTKs content, and a significant delay in the aging of the whole plant (Brugiere et al., 2008).

Currently, *PSAG12:IPT*, a carrier inducing *IPT* expression by the specific promoter of leaf senescence, has been transferred to a variety of grains and cash crops such as rapeseed, wheat, tomato, cauliflower, cotton, tobacco, and corn, and has shown to significantly delay the aging of leaves and other organs, affecting plant growth and development (Guo and Gan, 2011). PSAG12 drives *IPT* gene expression, increases cytokinin content, and delays leaf senescence. The increase of cytokinin in leaves can cause feedback into the inhibition of promoter activity and turn off the *IPT* gene, thus playing a role of self-regulation. The cytokinins synthesized by mature leaves of transgenic plants cannot be transported to other parts of the plant, thus they do not affect the normal development the plant, solving the problem of abnormal morphology of *IPT* constitutive overexpression transgenic plants. The construction of the chimeric gene *PSAG12:IPT* provides the possibility for the practical application of cytokinin biosynthesis related enzyme genes to regulate the senescence process of plant leaves (Liang et al., 2006).

Although the physiological function of CTK in delaying leaf senescence has been elucidated, the downstream molecular mechanisms of CTK in regulating leaf senescence is still not well understood. At present, it is generally believed that the CTK two-component system (TCS) pathway is involved in the regulation of CTK on leaf senescence. TCS is a multistep phosphate delivery system that includes histidine protein kinase (HKS) and downstream type A and type B response regulators (RR). The CTK response regulator (CRR) is located downstream of the signal and participates in the regulation of the physiological functions of CTK (Sakakibara, 2006). The first resolved downstream molecular mechanism of CTK regulating leaf senescence revealed that AHK3 played an important role in the CTK delay of leaf senescence. The functional deletion mutation of AHK3 delayed the CTK dependent leaf senescence and reduced the plants’ sensitivity to CTK, and removed the CTK dependent phosphorylation of the downstream response factor *ARR2* of type B CTK, thus regulating leaf senescence (Kim et al., 2006). *AHK2/AHK3* can be used as a receptor kinase combination to participate in the accumulation and regulation of plastid coding gene transcripts. The double mutant showed phenotypes such as fewer leaf cells and low chlorophyll content, both of which significantly affected development process (Riefler et al., 2006). In addition, *ARR16* could significantly induce leaf senescence through overexpression of type A CTK response factor, and the senescence process could not be inhibited by 6-BA, indicating that *ARR16* was involved in the CTK regulation of leaf senescence (Ren et al., 2009).

In maize kernels, CTKs levels peaked at approximately 10 days after pollination (DAP) (Brugiere et al., 2003). Our results revealed that the chlorophyll content, CTKs, and photosynthetic rate rose substantially throughout the development of transgenic plants. Additionally, the CTK content reached its maximum in the R1 period, which was consistent with previous reports, indicating that R1 is an important period for *ZmIPT2* to regulate CTKs, which in turn affected the later grain formation stage. Although CTKs content showed an overall decrease in the R6 period, transgenic strains still had a higher CTKs content than the receptor control. In the later stages of development, the leaves of the transgenic strains remained consistently green, and the yields of DNIPT2-C14, DNIPT2-C33, and DNIPT2-C34 were significantly higher than that of the receptor control. These results show that *ZmIPT2* gene could up-regulate the expression of CTKs content in the later stages of growth and development, improve the photosynthetic rate and chlorophyll content, and play a significant role in delaying leaf senescence, which were consistent with the results of previous studies.

### Overexpression of *ZmIPT2* gene improved grain yield in maize

Genetic improvement of grain yield is of great importance to insure food security. A large number of significant agronomic characteristics including yield showed continuous phenotypic variation. As a complex quantitative character, grain yield is determined many factors, such as grain number and grain weight (Melchinger et al., 1998; Yano, 2001). Various studies have proved that *IPT* gene has the function of improving crop yield and quality (Yang et al., 2017).

There were two types of *IPT*, one of which is an adenine modified tRNA, called tRNA IPT (EC. 2.5.1.8), which catalyzes the transfer of isopentenyl of dimethene diphosphate to the adenine residue of the precursor tRNA molecule to form a mature tRNA molecule. The modified nucleotide was located adjacent to the anti-codon, affecting the fidelity and efficiency of transcription. The other type of isopentyl transferase catalyzes the formation of IPMP, and is called *adenylate isopentenyl transferase (IPT: ec2.5.1.27*) which has been identified in *Agrobacterium tumefaciens* with a structure similar to tRNA IPT.

Studies based on ATP/ADP mutants and tRNA IPT showed that ATP/ADP IPTs are involved in the synthesis of the bulk of isopentenyladenine and *trans*-zeatin (t-Z) type CTK, while tRNA IPTs are required for *cis*-zeatin (cis-Z) type CTK production (Miyawaki et al., 2006). An *ATP/ADP-PpIPTs* study found that the overexpression of *PpIPT1*, *PpIPT3*, *PpIPT5*, and *PpIPT7* genes in *Arabidopsis* can increase cytokinin content in transgenic plants and improve salt resistance (Li et al., 2018). CRISPR/Cas9 targeted gene knockout *Phtheirospermum japonicum (Pj)IPT1a* inhibits parasite induced CTK response in host, revealing the important role of *PjIPT1a* in *Arabidopsis* phloem-induced CTK response (Greifenhagen et al., 2021). In the transgenic *Camellia sinensis* containing *IPT5*, the 3’ UTR variant 2 (3AS2) was found to be the main transcript which participated in the regulation of tea axillary bud germination and shoot branching, providing gene resources for improving the plant type of woody plants (Zhang et al., 2021). In addition, the triple mutant of *TaIPT8-5a/5b/5d* showed a decreased t-Z type CTK level and reduced drought tolerance under both normal and drought conditions. In contrast, drought induced *TaIPT8* transgenic wheat plants showed stronger drought tolerance, indicating that CTK plays a beneficial role in drought tolerance by regulating the redox state of cells (Wang et al., 2022).

Previous studies have elucidated the contribution of CTKs to crop yield (Jameson and Song, 2016; Chen et al., 2020). The specific role of *IPTs* in crop yield and its role in controlling grain production should be emphasized. Many efforts have been made to modify the spatial-temporal expression of *IPTs*, using promoters with different drivers to improve crop yield. For example, the *IPT* gene is responsible for delayed foliar senescence under the control of the SAG12 promoter (Gan and Amasino, 1995), which increased plant productivity (Kant et al., 2015). Plants that contained *IPT* driven by the SARK promoter had higher yield than the non-transgenic control (Leta et al., 2016).

Strategies for increasing yield should be focused on transgenic approaches based on the specific expression of *IPT* genes in early grain development (Sykorova et al., 2008). Cytokinin accumulation was increased in rice inflorescence meristems by decreasing the expression of the cytokinin degrading enzyme, cytokinin oxidase/dehydrogenase *(OSCKX2*), which in turn increased grain yield due to the increased number of reproductive organs (Ashikari et al., 2005). With the discovery of biosynthetic enzymes *IPT1* to *IPT10* (Irie et al., 2004; Brugiere et al., 2008; Vyroubalova et al., 2009) and degrading enzymes CKX1 to CKX12, the role of CTKs in maize kernel development has been made clearer (Morris et al., 1999; Brugiere et al., 2003; Massonneau et al., 2004; Smehilova et al., 2009; Vyroubalova et al., 2009). In *IPTs*, *IPT1*, and *IPT10* are abundantly constitutively expressed in all organs, but other IPT transcripts show different spatiotemporal expression patterns (Vyroubalova et al., 2009). In addition, another study showed that the modified cytokinin-degrading enzyme *Oslogl5* plants had significantly increased seed setting rate, total grains, fuller grains per panicle, and 1000-grain weight under drought conditions (Wang et al., 2020). Cheikh and Jones (1994) found that in the vegetative organs of maize, the content of CTKs varied with the expression level of the related gene. When the expression level of *ZmIPT2* reached the maximum, the cytokinin also reached the maximum, which showed that the expression level of *ZmIPT2* gene in the cell core was closely related to the content of cytokinin. In maize kernels, the endosperm, especially the basal metastatic cell layer (BETL), was the main expression site of *ZmIPT2* at 8–10 days after maize pollination, and its expression in BETL and endosperm continued until late development, suggesting that this gene plays an important role in CTK biosynthesis (Brugiere et al., 2008).

*In vitro* studies showed that *ZmIPT2*-thymine (T) with ADP, ATP, or AMP as substrate, had higher *IPT* activity than *ZmIPT2*-cytosine (C). It was found that a favorable *ZmIPT2*-T allele was related to grain weight (Weng et al., 2013). Yang recently reported that overexpression of *IPT2* gene in maize could regulate cytokinin content. Compared to the control, the photosynthetic rate and chlorophyll content of transgenic maize were significantly increased, delaying leaf senescence and increasing yield (Yang et al., 2017). These results indicated that regulating the metabolism of CTKs in reproductive organs may be an effective way to increase crop yield by increasing the flow of assimilates from source to sink or increasing sink capacity (Weng et al., 2013).

In addition, the high yield of crops has been shown to be closely related to the amount of source material on the plant. Leaves are the main source tissue for plants to carry out photosynthesis (Andrade et al., 2000). Therefore, crop yield can be roughly determined by the number of leaf sources, as well as the photosynthetic capacity of these leaf sources. With increasing age, leaf senescence also increases. By prolonging the functional period of leaves by delaying senescence, grain yield and plant biomass can be significantly improved (Dong et al., 2000; Thomas and Smart, 2010). Waggoner and Berger (1987) successfully used the functional period of green leaf area to express the correlation between maize leaf greenness and yield. However, some scholars believe that there is no obvious correlation between maize greenness and yield (Jiang et al., 2004; Massonneau et al., 2004; Wada and Wada, 2008).

This study discovered that the green maintenance capability of the leaves of the overexpression *ZmIPT2* strain after silking was positively correlated with the plot yield. The plot yield increased by an average of 12.49, 13.05, and 12.90%, respectively, compared to the yield of the receptor control strain. In this experiment, the *ZmIPT2* gene was transformed into maize to increase the yield, which was a rapid method when compared with other high-yield breeding methods. Three genetically stable T3 generation transgenic lines were obtained from this study. Our results showed that the overexpression of *ZmIPT2* gene could delay leaf senescence and significantly or extremely significantly improve yield traits such as ear length, ear diameter, 100 grain weight, and plot yield by significantly increasing plant CTKs content, net photosynthetic rate, and relative chlorophyll content. Among the transgenic lines, the DNIPT2-C14 line showed significantly increased yield traits when compared to the control, with a plot yield increase of 20.29% compared with the control. The field experiment was conducted for only 2 years: yield increase degree takes many years/field experiments to further confirm, so the results of this study, though promising, may be inaccurate. The three single-copy high-yield transgenic lines obtained in this will be screened out by backcross transfer method, and the target genes will be transferred into the backbone maize inbred lines for the preparation of hybrid combinations to breed new varieties of high-yield transgenic maize.

### Possible regulation mechanisms of *ZmIPT2* gene

The study of *IPT*, the main regulator of plant yield, provides important insights for crop breeding such as yield improvement by way of abiotic stress tolerance. Recent studies have shown that CYP735A1 and CYP735A2 are cytochrome P450 monooxygenases (P450), which catalyze the biosynthesis of t-Z (Takei et al., 2004). In *Arabidopsis*, it was found that that the co-expression of adenosine monopentenyltransferase AtIPT4 and CYP735A enabled yeast to excrete t-Z and nucleosides into the culture medium. Similarly, the *GRMZM2G022904* gene from this study is a cytochrome P450 (CYP450) superfamily protein, namely cytokinin hydroxylase, which can participate in the decomposition and anabolism of plant hormones and synthesis and metabolism of terpenoids, phenylpropanoids, alkaloids, sterols, and fatty acids in plants. Its mechanism of action in catalytic reaction is diverse and complex, and it is referred to as a universal catalyst (Schuler and Werck-Reichhart, 2003). The expression level of the *GRMZM2G022904* gene was positively correlated with the expression of *ZmIPT2* gene and positively regulated the *ZmIPT2* gene to participate in the *IPT* reaction and delay leaf senescence. However, our study only preliminarily verified that *GRMZM2G022904* was a key gene involved in the isopentenyl transferase pathway and the interaction between *ZmIPT2* and *GRMZM2G022904* by qRT-PCR. The relationship and mode of action between these two genes remains to be further studied.

## Data availability statement

The original contributions presented in this study are included in the article/Supplementary Material, further inquiries can be directed to the corresponding author/s.

## Author contributions

ZW and HD contributed to the conception and design of the work. YS, CL, and YZ performed the experiment. PG, QW, and LZ analyzed the data. YS and CL wrote the manuscript. All authors read and approved the final manuscript.

## Conflict of interest

The authors declare that the research was conducted in the absence of any commercial or financial relationships that could be construed as a potential conflict of interest.

## Publisher’s note

All claims expressed in this article are solely those of the authors and do not necessarily represent those of their affiliated organizations, or those of the publisher, the editors and the reviewers. Any product that may be evaluated in this article, or claim that may be made by its manufacturer, is not guaranteed or endorsed by the publisher.

## Footnotes

1
http://www.ncbi.nlm.nih.gov/gorf/gorf.html

2
https://npsa-prabi.ibcp.fr/cgi-bin/npsa_automat.pl?page=npsa_sopma.html

3
https://swissmodel.expasy.org/

4
http://www.ncbi.nlm.nih.gov/cdd

5
http://www.expasy.org/tools/protparam

6
http://web.expasy.org/protscale/

7
http://www.softberry.com/

8
http://bioinformatics.psb.ugent.be/webtools/plantcare/html/

9
https://cn.string-db.org/cgi/input?sessionId=bAHeTVEbwyfB&input_page_show_search=on

10
http://www.maizegdb.org/
