# Supplementary material for: Overexpression of ZmIPT2 gene delays leaf senescence and improves grain yield in maize
Source: Front Plant Sci. 2022 Jul 19;13:963873. doi: 10.3389/fpls.2022.963873 (PMC9344930; doi:10.3389/fpls.2022.963873)
Supplement: Supplementary file 1 [file Image_1.docx]

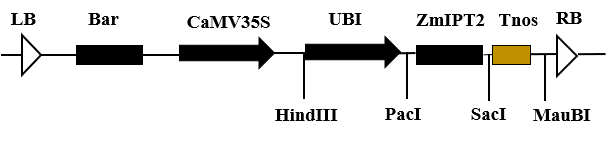


**Supplementary Figure 1. Schematic diagram of the expression vector pEC-Ubi-Tnos- *ZmIPT2* .** RB, right border; LB, left border; UBI, Ubiquitin promoter; Tnos, Nopaline synthase terminator; *ZmIPT2*, isopentenyl transferase 2 gene; Bar, Bialaphos resistance selectable marker gene; CaMV35S, Cauliflower mosaic virus 35S promoter. HindIII, PacI, SacI, and MauBI are restriction endonuclease recognition sites.
