# Supplementary material for: Overexpression of ZmIPT2 gene delays leaf senescence and improves grain yield in maize
Source: Front Plant Sci. 2022 Jul 19;13:963873. doi: 10.3389/fpls.2022.963873 (PMC9344930; doi:10.3389/fpls.2022.963873)
Supplement: Supplementary file 2 [file Image_2.docx]

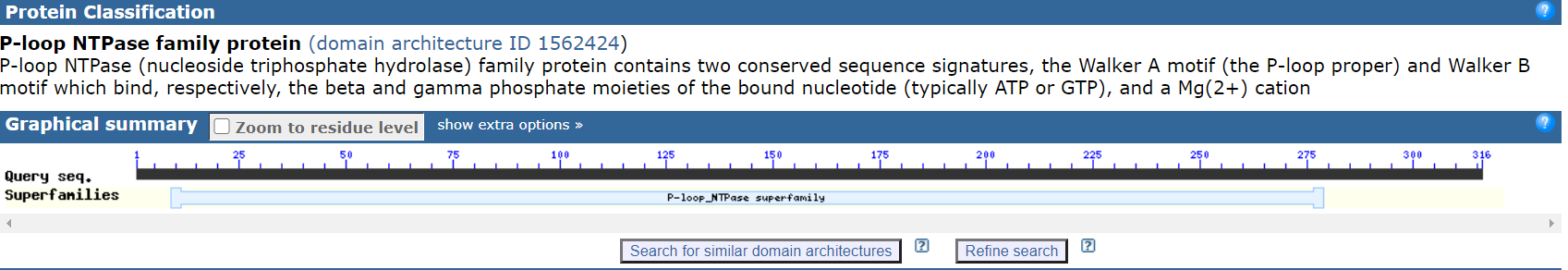


**Supplementary Figure2. Conserved domains of the ZmIPT2 protein predicted by CD-Search.** The light and dark gray rectangles indicate the major domains predicted by CD-Search, including a P-loop_NTPase super family.
