# Supplementary material for: Overexpression of ZmIPT2 gene delays leaf senescence and improves grain yield in maize
Source: Front Plant Sci. 2022 Jul 19;13:963873. doi: 10.3389/fpls.2022.963873 (PMC9344930; doi:10.3389/fpls.2022.963873)
Supplement: Supplementary file 3 [file Image_3.docx]

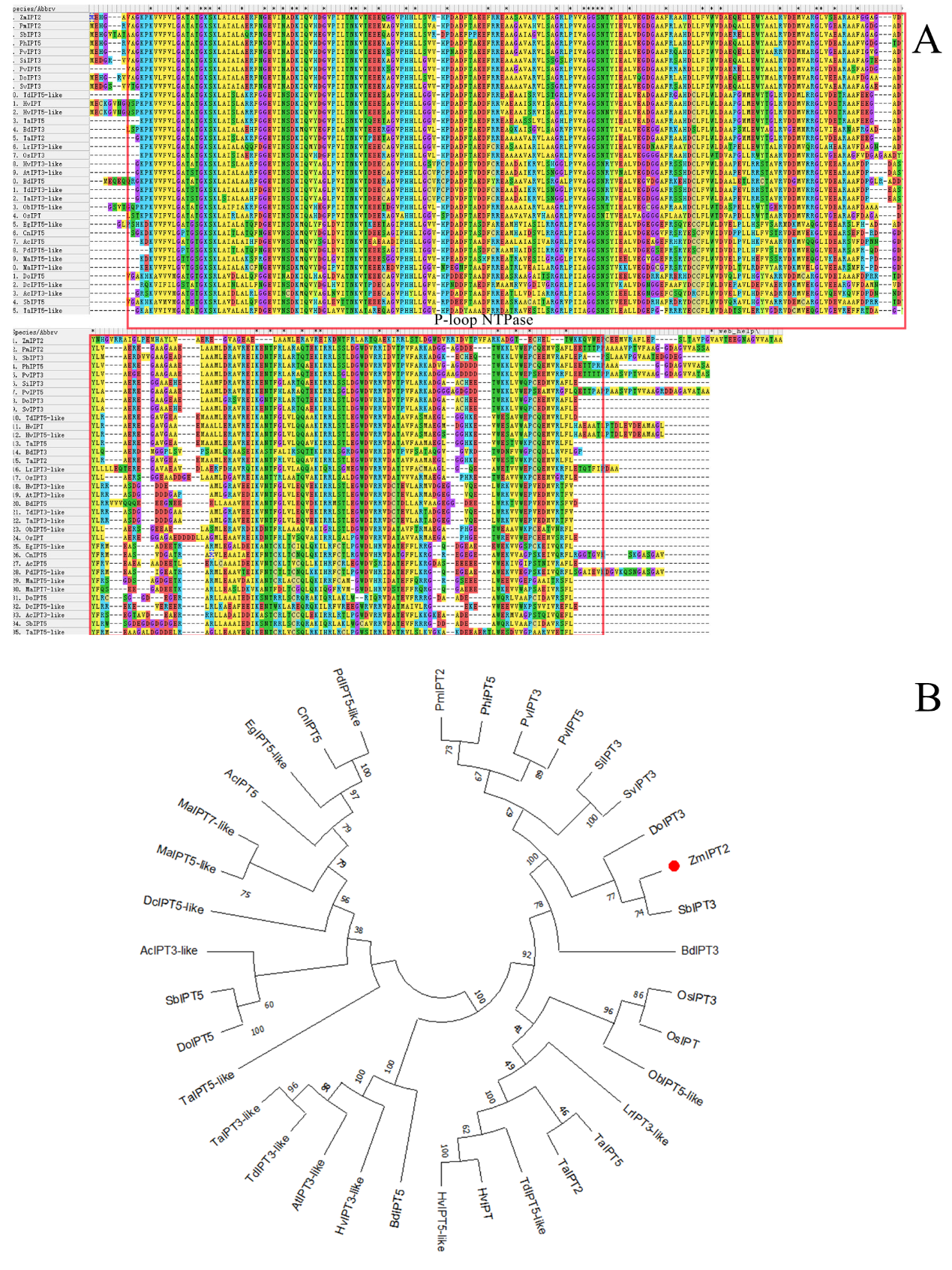


**SupplementaryFigure 3. Comparison of encoding amino acid sequence of ZmIPT2 protein with other plants. (A)** ClustalW was cuducted at Comparison of encoding amino acid sequence of ZmIPT2 protein with other plants. **(B)** Phylogenetic tree based on alignment of the deduced amino-acid sequences of ZmIPT2 and other known IPT proteins from different plant species. The scale bar represents conversion of branch length to genetic distance between clades (0.1 = 10% genetic distance).
