# Supplementary material for: Overexpression of ZmIPT2 gene delays leaf senescence and improves grain yield in maize
Source: Front Plant Sci. 2022 Jul 19;13:963873. doi: 10.3389/fpls.2022.963873 (PMC9344930; doi:10.3389/fpls.2022.963873)
Supplement: Supplementary file 4 [file Image_4.docx]

1CGTTCCTTAAACCAAATACACCACAAATTTCTTCAAAACAAACAAACACGATACATACTG

61GTTCTGTGCACAAAAAAGGCACGGACTGCTTCTTTTTCTATTTTTTGTTGTGTGCACAGA

121ATCGAGCGGCTACAATAATCAAGATCATCAAGACAATGGAGCACGGTGCCGTCGCCGGGA

M E H G A V A G

181AGCCCAAGGTGGTGTTCGTGCTCGGCGCCACAGCGACAGGGAAGTCGAAGCTCGCCATCG

K P K V V F V L G A T A T G K S K L A I

241CCCTCGCCGAGCGCTTCAACGGTGAGGTTATCAACGCTGACAAAATCCAGGTCCACGATG

A L A E R F N G E V I N A D K I Q V H D

301GCGTGCCCATCATCACGAACAAGGTCACAGAGGAAGAGCAGGGCGGGGTGCCCCACCACC

G V P I I T N K V T E E E Q G G V P H H

361TGCTCAGCGTCCGCCACCCGGACGCCGACTTCACTGCGGAGGAGTTCCGACGTGAGGCGG

L L S V R H P D A D F T A E E F R R E A

421CCAGCGCCGTGGCCCGCGTGCTCTCGGCGGGCCGCCTCCCCGTCGTGGCAGGCGGGTCCA

A S A V A R V L S A G R L P V V A G G S

481ACACCTACATCGAGGCACTGGTGGAAGGCGACGGCGCCGCCTTCCGCGCGGCGCACGACC

N T Y I E A L V E G D G A A F R A A H D

541TCCTCTTCGTCTGGGTGGACGCGGAGCAGGAGCTGCTGGAGTGGTACGCCGCGCTGCGCG

L L F V W V D A E Q E L L E W Y A A L R

601TGGACGAGATGGTGGCCCGCGGGCTGGTGAGCGAGGCTCGCGCGGCGTTCGGCGGCGCCG

V D E M V A R G L V S E A R A A F G G A

661GGGTTGACTACAACCATGGCGTGCGCCGCGCCATCGGCCTGCCGGAGATGCACGCCTACC

G V D Y N H G V R R A I G L P E M H A Y

721TGGTGGCGGAGCGCGAGGGCGTCGCTGGGGAGGCCGAGCTCGCGGCCATGCTGGAACGCG

L V A E R E G V A G E A E L A A M L E R

781CGGTGCGCGAGATCAAGGACAACACCTTCCGCCTCGCGCGCACGCAGGCGGAGAAGATCC

A V R E I K D N T F R L A R T Q A E K I

841GGCGCCTCAGCACGCTCGACGGCTGGGACGTCCGCCGCATCGACGTGACCCCCGTGTTCG

R R L S T L D G W D V R R I D V T P V F

901CGCGCAAGGCCGATGGCACTGAGTGCCACGAGCTGACTTGGAAGAAGCAGGTGTGGGAGC

A R K A D G T E C H E L T W K K Q V W E

961CGTGCGAGGAGATGGTGAGGGCTTTCCTCGAGCCGTCCCTGACTGCCGTTCCAGGTGTTG

P C E E M V R A F L E P S L T A V P G V

1021CAGTAACTGAAGAAGGGAACGCCGGCGTCGTCGCTACTGCTGCACCCGCTGGTGATGTCG

A V T E E G N A G V V A T A A P A G D V

1081TCGTCCCAACTGGCGATGTCGTCACCGCCGTGGCTGATGCATAAGTAGCTAGCGGACGTA

V V P T G D V V T A V A D A *

1141GCGCATGCATGCAATGCATGCAGGCTGGCTGGCTGGCTTAATTAGTGCCTCCGACTTGCT

1201TTAAACTCATGTAGCTGCGTCCATGGGAGAGGGTGAGATACAAGTTTATGCGACTTATAT

1261TTCTTTCTAAATTTAAATGGATCTCGGATCCGTAGTATCTGGTTTAATATAATTATAATA

1321TTTCCTTCGAATTATTATA

**Supplementary Figure 4. Full-length cDNA sequence and encoding amino acid sequence of *ZmIPT2* .**

Frame means initiation codon and * means termination codon.
