# Supplementary material for: Overexpression of ZmIPT2 gene delays leaf senescence and improves grain yield in maize
Source: Front Plant Sci. 2022 Jul 19;13:963873. doi: 10.3389/fpls.2022.963873 (PMC9344930; doi:10.3389/fpls.2022.963873)
Supplement: Supplementary file 5 [file Image_5.docx]

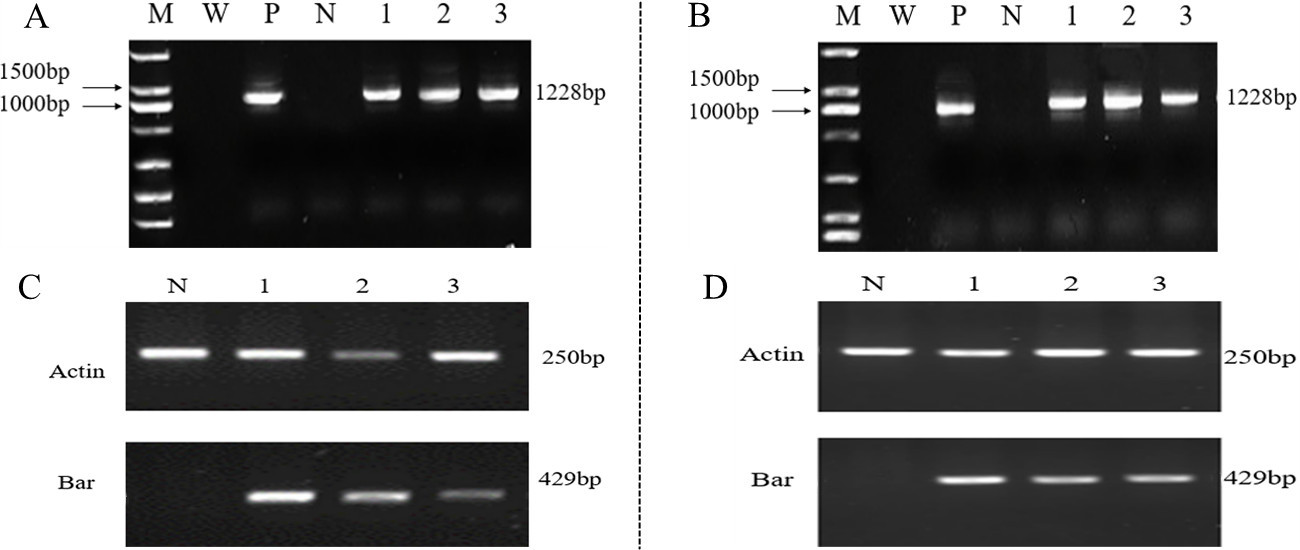


**Supplementary Figure 5. Molecular detection of transgenic maize lines with *ZmIPT2* gene. (A)**,**(B)** were T2 and T3 PCR analysis of transformed plants using *ZmIPT2* primers. M: Maker(DL2000 PLUS); W: Water control; P: Positive plasmid; Bar proteinin T2 and T3 transgenic lines by colloidal gold strip test N: Negative control; 1-3: DNIPT2-C14,DNIPT2-C33 and DNIPT2-C34. **(C)**,**(D)** were T2 and T3 RT-PCR analysis of transformed samples using Bar gene-specific primers. A 439-bp PCR product was amplified with Bar gene-specific primers from three transgenic maize lines (DNIPT2-C14,DNIPT2-C33 and DNIPT2-C44) by reverse transcriptase-polymerase chain reaction (RT-PCR).
