# Supplementary material for: Overexpression of ZmIPT2 gene delays leaf senescence and improves grain yield in maize
Source: Front Plant Sci. 2022 Jul 19;13:963873. doi: 10.3389/fpls.2022.963873 (PMC9344930; doi:10.3389/fpls.2022.963873)
Supplement: Supplementary file 6 [file Image_6.docx]

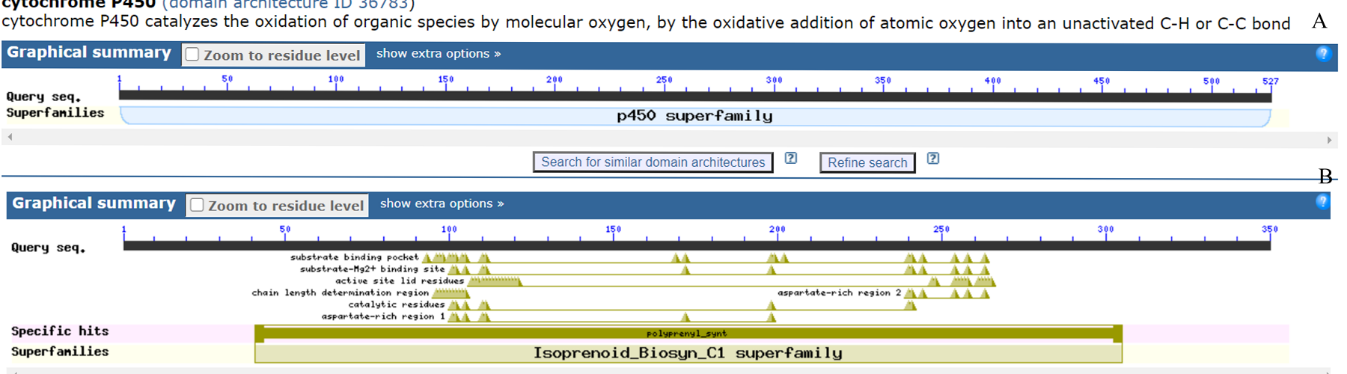


**Supplementary Figure 6. Predicted interacting gene protein domain.**(A) *GRMZM2G022904* gene protein domain, (B) *GRMZM2G168681* and *GRMZM2G147721* gene protein domain.
