# Supplementary material for: Overexpression of ZmIPT2 gene delays leaf senescence and improves grain yield in maize
Source: Front Plant Sci. 2022 Jul 19;13:963873. doi: 10.3389/fpls.2022.963873 (PMC9344930; doi:10.3389/fpls.2022.963873)
Supplement: Supplementary file 7 [file Table_1.docx]

**Table 1 Prediction subcellular localization of ZmIPT2 protein**

| Destination of cell compartment | Certainty |
| --- | --- |
| nuclear | 0.304 |
| cytoplasmic | 0.478 |
| mitochondrial | 0.174 |
| endoplasmic reticulum | 0.044 |
