# Supplementary material for: Overexpression of ZmIPT2 gene delays leaf senescence and improves grain yield in maize
Source: Front Plant Sci. 2022 Jul 19;13:963873. doi: 10.3389/fpls.2022.963873 (PMC9344930; doi:10.3389/fpls.2022.963873)
Supplement: Supplementary file 8 [file Table_2.docx]

**Table 2 Primer sequences**

| Primers | Sequences (5'→3') | Amplified region |
| --- | --- | --- |
| KB-*ZmIPT2*-F | TAGCCCTGCCTTCATACGC | *ZmIPT2* copy number analysis |
| KB-*ZmIPT2*-R | CGAGCACGAACACCACCTT | *ZmIPT2* copy number analysis |
| Actin-F | GTTGGGCGTCCTCGTCA | Actin1 gene |
| Actin-R | TGGGTCATCTTCTCCCTGTT | *Actin1* gene |
| qRT-PCR-F | CATCATCACGAACAAGGTCACA |  |
| qRT-PCR-R | CAACTCCTCCGCAGTGAAGT |  |
| INqRT-*Zm904*-F | CCCGACAAGCTGAAGGGAC | *ZmIPT2* interaction gene |
| INqRT-*Zm904*-R | CCGACGCTGCATCCTGAA | *ZmIPT2* interaction gene |
| INqRT-*ZmFps1*-F | GTGTTCCTGGCTAGTCGTGC | *ZmIPT2* interaction gene |
| INqRT-*ZmFps1*-R | CCTGGAAAACCTCCTCCAGTT | *ZmIPT2* interaction gene |
| INqRT-*ZmFps2*-F | GAACTTGACCTAGAGGCGGT | *ZmIPT2* interaction gene |
| INqRT-*ZmFps2*-R | TTGGCTGGGCTTCAATGTCT | *ZmIPT2* interaction gene |
